# Spectroscopic and Chromatographic Characterization of Two Isomeric Cathinone Derivatives: *N*-Butyl-Norbutylone and *N*-Ethylhexylone

**DOI:** 10.3390/molecules30102182

**Published:** 2025-05-16

**Authors:** Marcin Rojkiewicz, Piotr Kuś, Josef Jampilek, Andrzej Bąk, Violetta Kozik

**Affiliations:** 1 Institute of Chemistry, University of Silesia, 9 Szkolna Street, 40-006 Katowice, Poland; pkus@ich.us.edu.pl (P.K.); josef.jampilek@gmail.com (J.J.); andrzej.bak@us.edu.pl (A.B.); 2 Department of Analytical Chemistry, Faculty of Natural Sciences, Comenius University, Ilkovicova 6, 842 15 Bratislava, Slovakia

**Keywords:** new psychoactive substances, *N*-butyl-norbutylone, *N*-ethylhexylone, mass spectrometry, NMR spectroscopy

## Abstract

In this study, two isomeric cathinone derivatives, *N*-butyl-norbutylone and *N*-ethylhexylone, seized on the illicit drug market in Poland, were described and characterized by various instrumental analytical methods. The compounds were characterized by electrospray ionization mass spectrometry, high-resolution mass spectrometry, gas chromatography–mass spectrometry, and nuclear magnetic resonance spectroscopy. The two investigated compounds were confirmed as 1-(2*H*-1,3-benzodioxol-5-yl)-2-(butylamino)butan-1-one and 1-(2*H*-1,3-benzodioxol-5-yl)-2-(ethylamino)hexane-1-one, both of which were cathinone derivatives available on the new psychoactive substances (NPS) market. The obtained analytical data should be useful for forensic and toxicological purposes for quick and reliable compound identification.

## 1. Introduction

In recent years, the global drug market has experienced a significant increase in the availability and consumption of new psychoactive substances (NPS), among which, synthetic cathinones play a significant role [1]. These substances are synthetic analogs of cathinone, the psychoactive component of the khat plant (*Catha edulis*), and have gained popularity due to their stimulant and empathogenic effects, which are similar to those of amphetamines and MDMA (3,4-methylenedioxymethamphetamine) [2,3].

Synthetic cathinones have become key components of so-called “legal highs” or “bath salts” and are often promoted as substitutes for traditional stimulants. Their widespread presence in the illicit drug market can be attributed to their relatively straightforward synthesis, broad structural variability, and the ease with which they evade legal regulations through minor chemical modifications [4]. Therefore, cathinones emerging in the NPS market pose significant challenges for law enforcement, forensic experts, and healthcare professionals.

Synthetic cathinones first gained international attention in the early 2000s, with mephedrone (4-methylmethcathinone) being among the most widely abused substances [5]. Following legislative restrictions on mephedrone in several countries, clandestine laboratories began synthesizing new derivatives, including methylone, butylone, pentylone, and eutylone, which possess similar psychoactive properties [6]. These compounds are often sold online in head shops or through illicit street markets and are frequently mislabeled as “research chemicals” or “not for human consumption” to evade regulation.

Market surveys indicate that synthetic cathinones are among Europe and North America’s most commonly detected NPS. According to the European Monitoring Centre for Drugs and Drug Addiction (EMCDDA), cathinones account for approximately 25% of all of the new synthetic drugs identified in forensic laboratories over the past decade. Despite increasing regulatory measures, their popularity is attributed to their potent stimulant effects, affordability, and widespread availability [7,8].

Cathinones belong to the broader class of phenethylamines and share a core structure with them. Compared to them, cathinones possess an alkyl group attached to the α carbon and a keto moiety at the β carbon of the phenylethyamine core. The presence of the β-keto group distinguishes them from amphetamines and contributes to their unique pharmacokinetic and pharmacodynamic properties. Synthetic cathinones are commonly categorized based on their structural modifications:

- *N*-Alkylated cathinones: Includes mephedrone, pentedrone, and 4-methylethcathinone (4-MEC), which exhibit stimulant and empathogenic properties.
- Methylenedioxy-substituted cathinones: Includes methylone, butylone, and pentylone, which share structural similarities with MDMA and exert serotonergic effects.
- Pyrrolidine-containing cathinones: Includes α-pyrrolidinovalerophenone (α-PVP) and methylenedioxypyrovalerone (MDPV), which act as potent dopamine reuptake inhibitors and exhibit vigorous psychostimulant activity.

Among these classes, methylenedioxy-substituted cathinones (e.g., methylone and butylone) are particularly notable for their MDMA-like effects, making them attractive substitutes for traditional entactogens [9,10,11,12,13].

Synthetic cathinones primarily affect the monoaminergic system by altering the activity of dopamine (DA), norepinephrine (NE), and serotonin (5-HT) transporters. Their mode of action closely resembles that of amphetamines and MDMA, though slight structural differences lead to variations in their potency, duration of effects, and subjective experiences.

Methylenedioxy-substituted cathinones, such as methylone and butylone, closely resemble MDMA in their pharmacological action. These compounds act as both releasers and reuptake inhibitors of serotonin, leading to increased synaptic 5-HT levels, which are associated with their empathogenic and entactogenic effects. However, compared to MDMA, these cathinones often exhibit shorter durations of action and reduced potency in serotonin release [14,15,16,17].

**Figure 1:**
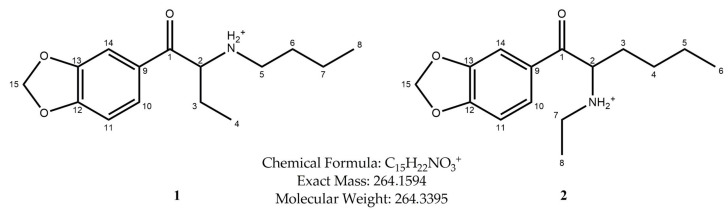
Structures of *N*-protonated *N*-butyl-norbutylone (**1**) and *N*-ethylhexylone (**2**).

In this study, we present the identification and physicochemical characterization of two isomeric cathinone MDMA-like derivatives, namely, *N*-butyl-norbutylone (**1**) (1-(2*H*-1,3-benzodioxol-5-yl)-2-(butylamino)butan-1-one) and *N*-ethylhexylone (**2**) (1-(2*H*-1,3-benzodioxol-5-yl)-2-(ethylamino)hexane-1-one), found in seized materials (shown in Figure 1).

The data for characterizing the substances were obtained by gas chromatography–mass spectrometry (GC-MS), direct infusion electrospray ionization mass spectrometry (ESI-MS), high-resolution mass spectrometry (HR-MS), and nuclear magnetic resonance spectroscopy (NMR). To the best of our knowledge, this is the first report that identifies in the seized material and characterizes, in detail, *N*-ethylhexylone circulated on the NPS market; however, some data on *N*-butyl-norbutylone have already been published [18].

## 2. Results

**Figure 2:**
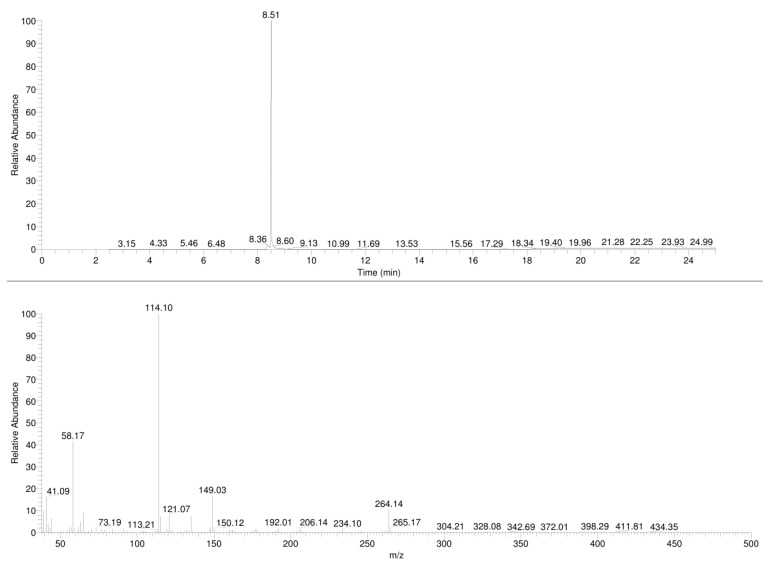
GC-EI-MS spectra of *N*-butyl-norbutylone (**1**).

**Figure 3:**
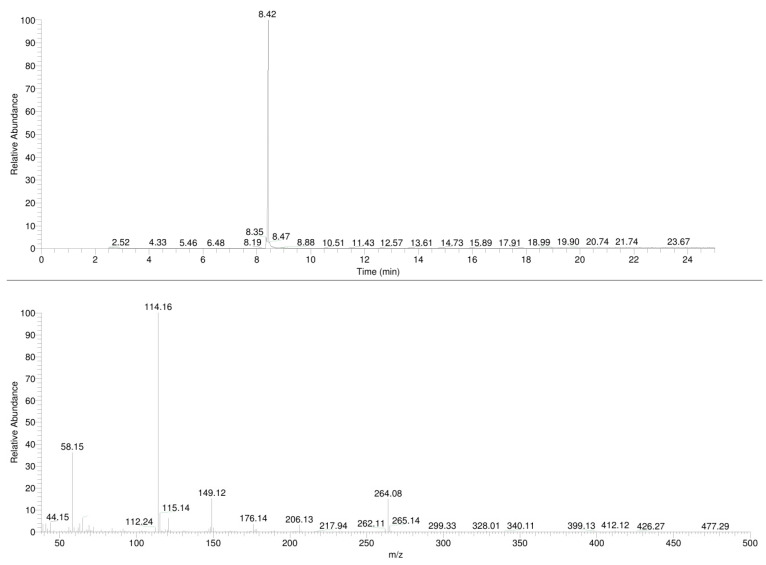
GC-EI-MS spectra of *N*-ethylhexylone (**2**).

The samples were analyzed by gas chromatography with mass spectrometric detection (GC-MS), and the resulting mass spectra and chromatograms of the compounds **1**,**2** are shown in Figure 2 and Figure 3, respectively.

In the ESI-MS spectrum, the protonated molecule [M + H^+^] was seen at 264 *m*/*z* for both investigated compounds. The samples were directly infused to the ion source and were also analyzed in the MS/MS mode.

The investigated compounds were also analyzed by HR-MS by direct infusion, which yielded the protonated molecule at 264.1602 *m*/*z* (C_15_H_22_NO_3_, calculated as 264.1600 *m*/*z*; mass: accuracy 0.8 ppm) for compound **1**, and at 264.1597 *m*/*z* (C_15_H_22_NO_3_, calculated as 264.1600 *m*/*z*; mass accuracy: 1.1 ppm) for compound **2**.

**Figure 4:**
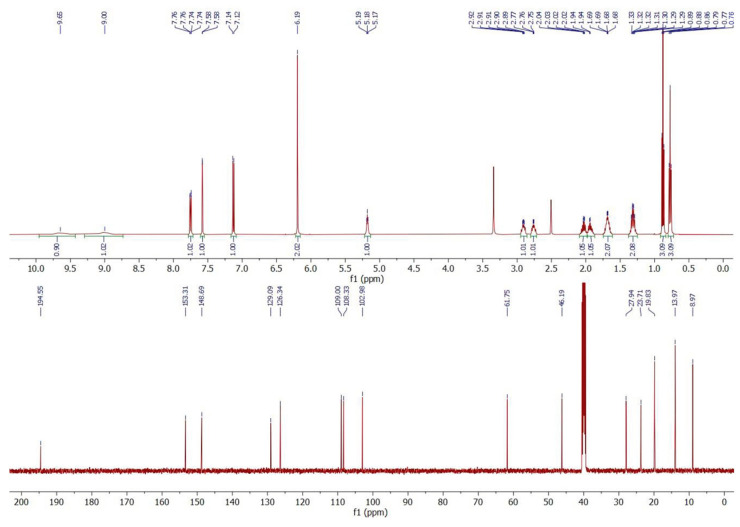
^1^H (upper) and ^13^C (lower) nuclear magnetic resonance spectra of *N*-butyl-norbutylone (**1**).

**Figure 5:**
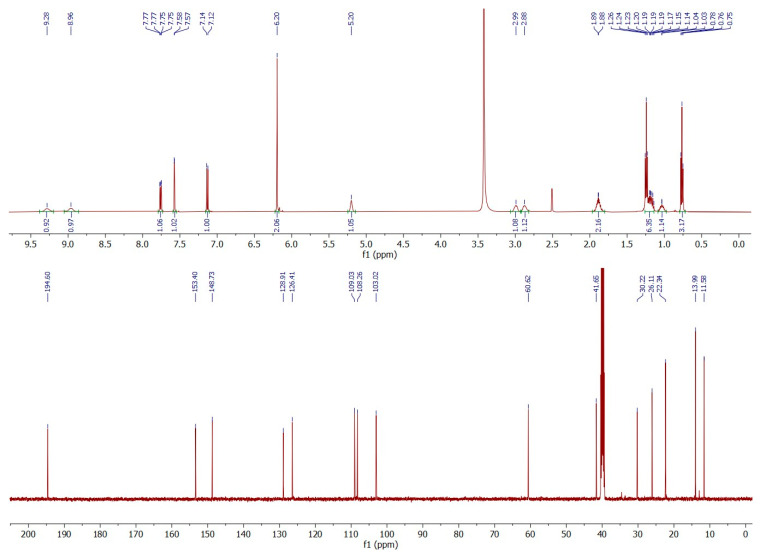
^1^H (upper) and ^13^C (lower) nuclear magnetic resonance spectra of *N*-ethylhexylone (**2**).

NMR was employed to confirm the structures of investigated compounds. The ^1^H and ^13^C nuclear magnetic resonance spectra for both compounds are shown in Figure 4 and Figure 5. Furthermore, all data concerning chemical shifts are shown in Table 1 and Table 2.

## 3. Discussion

### 3.1. GC-MS

**Figure 6:**
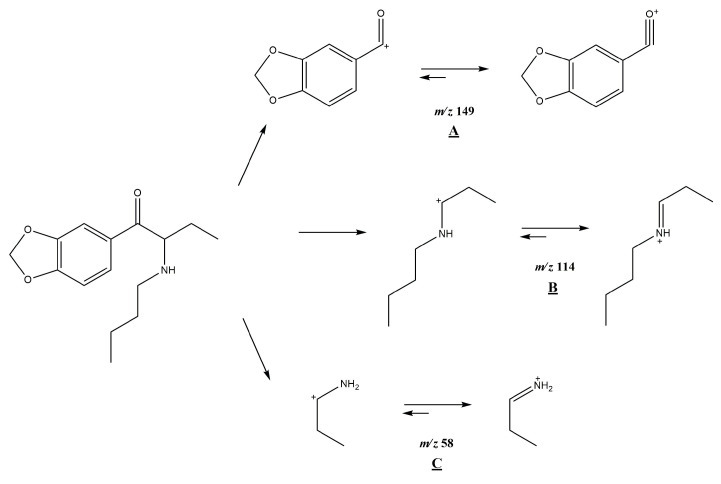
Predicted EI-MS fragmentation pathway of *N*-butyl-norbutylone (**1**).

**Figure 7:**
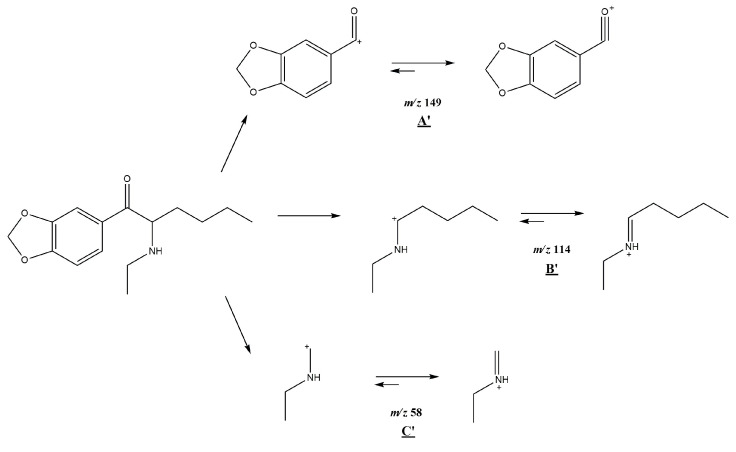
Predicted EI-MS fragmentation pathway of *N*-ethylhexylone (**2**).

In the mass spectrum obtained in the electron impact ionization mode (EI-MS), shown in Figure 1 and Figure 2, one main fragment ion was detected at 114 *m*/*z* for both investigated compounds. Other less intense fragments present in the spectra were at 58 and 149 *m*/*z* for compound **1** and for compound **2**. Possible structures of the fragmentation products derived from the parent structure of the analyzed compounds are presented in Figure 6 and Figure 7, respectively.

The fragmentation of both investigated compounds is consistent with the fragmentation pathways proposed in the literature [19]. The most intense fragment ions in GC-MS are the ions detected at 114 *m/z*, and this suggests that the bond cleavage was between carbon C1 and C2 (carbon numbering shown in Figure 1) for both investigated compounds. When compared with the fragmentation of other cathinone derivatives, it can be noticed that the observed α cleavage is the most common bond cleavage for cathinones in the EI mode.

If the fragmentation pathways of the analyzed compounds are analyzed (see Figure 6 and Figure 7), it can be observed that despite the various possible structures of the fragment ions (excluding structures A and A’), and due to the identical *m*/*z* values of the main peaks (structures B; B’ and C; C’), it is difficult to unambiguously determine which compound is present in the sample, based solely on the EI-MS spectra. It is also difficult to definitely distinguish the investigated compounds based on retention time, as shown in the chromatograms in Figure 2 and Figure 3; the retention times of the compounds are very similar and remain so despite changes in analytical conditions. While it is possible to improve the separation of closely related compounds by selecting a different chromatographic column, it must be noted that most forensic laboratories routinely use HP-5 type columns (or their equivalents) for the analysis of psychoactive substances. Taking this into consideration, it must be stated that in the case of analyzing controlled substances seized by law enforcement, relying solely on GC-MS analysis (using the most commonly used general-purpose columns) may result in misleading or inaccurate conclusions.

### 3.2. ESI-MS

As noted in the ESI-MS spectrum, the protonated molecule [M + H^+^] was seen at *m*/*z* 264 for both investigated compounds. Since the mass spectrometry analysis of the organic compounds, with gentle ionization techniques like electrospray ionization, is used, providing poor information about the structure of the compound, the samples were analyzed in the MS/MS mode as well.

**Figure 8:**
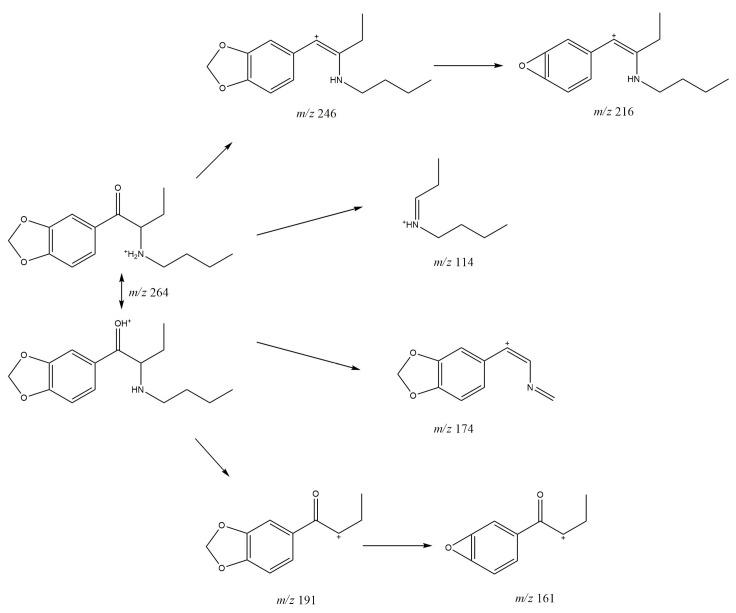
Predicted ESI-MS/MS fragmentation pathway of *N*-butyl-norbutylone (**1**).

**Figure 9:**
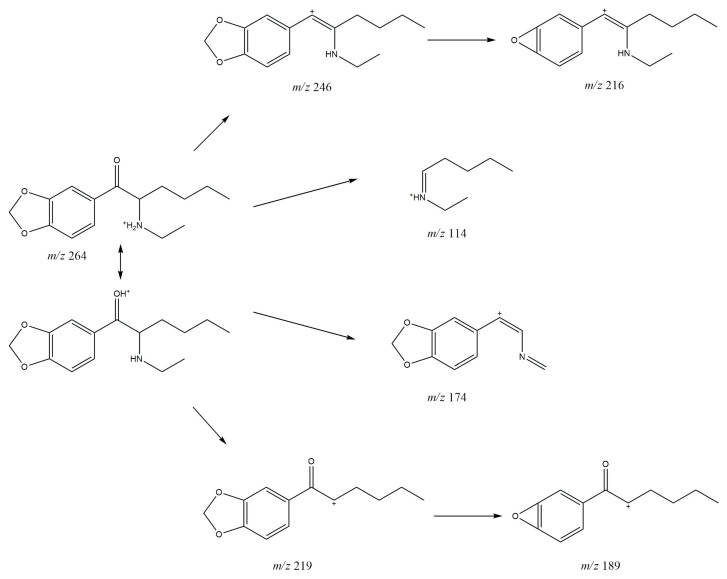
Predicted ESI-MS/MS fragmentation pathway of *N*-ethylhexylone (**2**).

In the MS/MS mod, the elimination of the water molecule [M + H^+^ − H_2_O] was observed at 246 *m*/*z* for both of the investigated compounds, which is characteristic of certain cathinone derivatives [20,21]. The probable fragmentation pathways for compounds **1** and **2** are presented in Figure 8 and Figure 9, respectively, which is in line with the fragmentation pathways proposed in the literature [18]. The ESI-MS/MS spectra for the investigated compounds are available in Supplementary Material (Figures S1 and S2).

Analysis of the ESI-MS/MS spectra reveals that compounds **1** and **2** exhibit the same peaks at 246, 216, 174, and 114 *m*/*z*. Therefore, the presence of these peaks does not allow for differentiation between the analyzed compounds. However, considering that peaks at *m*/*z* 191 and 161 are present for compound **1**, and peaks at 219 and 189 *m*/*z* are observed for compound **2**, it can be concluded that using the ESI-MS/MS technique enables clear differentiation between the studied compounds. It should be noted that chromatography (both gas and liquid) and mass spectrometry are the most commonly used analytical techniques in forensic laboratories.

### 3.3. ^1^H and ^13^C NMR

NMR spectroscopy was employed to confirm the structures of the investigated compounds. The data for compounds **1** and **2** are presented in Table 1 and Table 2, respectively. Additionally, the ^1^H-^1^H NMR spectra for the investigated compounds are available in electronic Supplementary Material (Figures S3 and S4).

In the ^1^H NMR spectrum of compound **1**, characteristic triplets originating from the methyl groups C4 and C8 protons are recorded at δ = 0.77 ppm and δ = 0.88 ppm, respectively. Additionally, the ^1^H-^1^H NMR spectrum indicates that the C4 protons couple only with the methylene C3 protons, while the C8 protons couple with the two C7 protons. Moreover, it can be observed that the C2 protons couple with the C3 protons, which indicates that the ethyl group is attached to the carbon in position 2. Further analysis of the multiplicity of signals in the ^1^H NMR spectrum for C5–C7 protons, as well as their interactions reported in the ^1^H-^1^H NMR spectrum, confirms that the butyl group is attached to the nitrogen atom. In the aromatic region, interactions between the C10 and C11 protons are recorded, while the C14 protons show no interactions. Also, no coupling interactions occur for the methylene group C15 protons.

The signals in the ^1^H NMR spectrum of compound **1** are well separated, even in the aliphatic region, while for compound **2**, the signals of C4, C5, and C8 protons appear very close; however, the signal arrangement is similar to that observed for compound **1**. So, in the ^1^H NMR spectrum of compound **2**, two triplets of the C6 and C8 methyl groups are recorded at δ = 0.76 ppm and δ = 1.24 ppm, respectively. Analysis of the proton interactions in ^1^H-^1^H NMR spectrum, as well as the multiplicity in the ^1^H NMR signals, confirms that the butyl group is attached to the C2 methine carbon and the ethyl group is bonded to the nitrogen atom, i.e., the opposite of compound **1**. The interactions of aromatic C10 and C11 protons are also observed in compound **2**, while no interactions occur for C14 and C15 protons. This suggests that the 1,3-benzodioxol-5-yl moiety is common for both investigated compounds and that both are isomers.

Based on the NMR and mass spectrometry analysis, the structure of the investigated compounds was confirmed. However, considering their structural similarity, it must be stated that the use of only the 1D NMR technique is insufficient to elucidate the structures of the compounds. The analyzed compounds have practically identical signals in the aromatic region of the NMR spectra, both in the proton and carbon spectra. Also, the aliphatic region of the spectra does not allow for the distinction of those two compounds. Only the application of additional techniques, such as chromatography and mass spectrometry, as well as the analysis of 2D NMR spectra, allows for the differentiation of compounds **1** and **2**.

## 4. Materials and Methods

### 4.1. Chemicals

The methanol (Sigma-Aldrich, Poznan, Poland) used for analysis was of the HPLC purity grade. The deuterated dimethylsulfoxide (DMSO-*d*_6_) for NMR analysis was purchased from Sigma-Aldrich.

### 4.2. Sample Preparation

The samples were provided by drug enforcement agencies as examples of materials seized on the illicit drug market, and both of them were in pure powdered form. For analysis purposes, there was no need to purify the samples. For gas chromatography and electrospray ionization mass spectrometry, 10 mg of each sample was dissolved in methanol (1 mL) without the need for ultrasonication. An aliquot of 10 µL was collected from the solution and diluted one hundred-fold with methanol and analyzed by GC-MS and ESI-MS. For NMR spectroscopic analysis, 10 mg of each sample was dissolved in 0.6 mL DMSO-*d*_6_.

### 4.3. Gas Chromatography Mass Spectrometry (GC-MS) Analysis

For GC-MS analysis, the Thermo Trace Ultra chromatograph was used, coupled with the Thermo ITQ900 mass spectrometer (Thermo Scientific, Warsaw, Poland). The analyses were carried out with the use of the Rxi^®^-5Sil MS column (Restek, Bellefonte, PA, USA). The following working parameters were employed: injector temperature, 260 °C; oven temperatures, 100 °C for 2 min, ramping at 20 °C/min to 260 °C; carrier gas (helium) flow rate, 1.5 mL/min; MS transfer line temperature, 250 °C; MS source temperature, 250 °C; injection volume, 1 μL; split mode, 1:50.

### 4.4. Direct Infusion Electrospray Ionization Mass Spectrometry (ESI-MS)

A Thermo TSQ Vantage mass spectrometer with an electrospray ionization source (Thermo Scientific, Warsaw, Poland) was used. The following working parameters for the direct infusion ESI-MS experiment were employed: sheath gas pressure, 5 psi; H-ESI vaporizer temperature, 50 °C; spray voltage, 3500 V; ion transfer tube temperature, 50 °C; direct infusion syringe flow rate, 5 μL/min. The obtained data were processed using Xcalibur and TSQTune software version 2.1 (Thermo Scientific). The analytes were electrosprayed in the positive mode (ESI(+)-MS). Fragmentation in the ESI-MS/MS mode was carried out in the scanning range of 50–270 *m*/*z* for both compounds. The ESI-carrier and collision gases were nitrogen and argon, respectively.

### 4.5. High-Resolution Mass Spectrometry (HR-MS)

Mass spectrometry analyses were performed using Ultra-Performance Liquid Chromatograph ACQUITY UPLC I-Class (Waters Corp., Milford, MA, USA) coupled with a Synapt G2-S mass spectrometer (Waters) equipped with the electrospray ion source and a quadrupole-time-of-flight mass analyzer. The resolving power of the TOF analyzer was set to 20,000 FWHM. The instrument was controlled, and recorded data were processed using the MassLynx V4.1 software package (Waters). The mass spectrometry measurements were performed in a positive mode. The measurements in the positive mode were performed with a capillary voltage set to 3.00 kV. The desolvation gas flow was 700 L/h, and the temperature was set to 300 °C. The sampling cone voltage and source offset were set to 20 V, and the source temperature was 120 °C. The sample was dissolved in methanol and injected directly into the electrospray ion source. The instrument worked with external calibration on sodium formate in the mass range of 50–1200 *m*/*z*. The leucine–enkephalin solution was used as the lock–spray reference material. The lock–spray spectrum of the leucine–enkephalin was generated by the lock–spray source, and correction was undertaken for every spectrum. The exact mass measurements for all peaks were performed within a 3 mDa mass error.

### 4.6. NMR Spectroscopy

The NMR spectra of the samples were recorded with the use of the UltraShield 400 MHz apparatus (Bruker, Bremen, Germany), and deuterated dimethylsulfoxide (DMSO-*d*_6_) was used as a solvent. The data were collected with the chemical shift referenced to a residual solvent signal.

## 5. Conclusions

In this study, we presented chromatographic and spectroscopic characterization of two isomeric cathinone derivatives *N*-butyl-norbutylone (**1**) and *N*-ethylhexylone (**2**), which are available on the NPS market. The presented study is a comprehensive characterization of the compounds and can be helpful for forensic analytical chemists and toxicologists. As already mentioned, chromatographic methods and mass spectrometry are routinely used in forensic laboratories. This work has shown that the use of only the GC-MS technique to analyze the investigated compounds is insufficient due to their similar EI fragmentation patterns. However, analyzing the fragmentation of the studied compounds using the ESI-MS/MS technique allows for their differentiation. Additionally, it enables the selection of ion pairs for quantitative analysis in the selected reaction monitoring (SRM) mode. It also has to be noted that there may be many more isomeric cathinones, so the methodology described in this work may be helpful in their identification. To the best of our knowledge, this study provides the first comparative analysis of those two isomeric cathinone derivatives and provides the first detailed and comprehensive analytical data for *N*-ethylhexylone.

## Figures and Tables

**Table 1:** ^1^H and ^13^C nuclear magnetic resonance data for *N*-butyl-norbutylone (**1**).

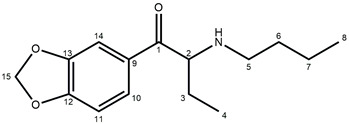		
Atom Position	Carbon Chemical Shifts (ppm)	Proton Chemical Shifts (ppm)
1	194.55	–
2	61.75	5.18 (t, *J* = 5.4 Hz, 1H)
3	23.71	1.94 (m, 1H); 2.02 (m, 1H)
4	8.97	0.77 (t, *J* = 7.5 Hz, 3H)
5	46.19	2.76 (m, 1H); 2.91 (m, 1H)
6	27.94	1.68 (m, 2H)
7	19.83	1.31 (m, 2H)
8	13.97	0.88 (t, *J* = 7.4 Hz, 3H)
9	129.09	–
10	126.34	7.75 (dd, J = 8.2; 1.8 Hz, 1H)
11	109.00	7.13 (d, *J* = 8.2 Hz, 1H)
12	153.31	–
13	148.69	–
14	108.33	7.58 (d, *J* = 1.8 Hz, 1H)
15	102.98	6.19 (s, 2H)
N-H	–	9.00 (bs, 1H); 9.65 (bs, 1H)

**Table 2:** ^1^H and ^13^C nuclear magnetic resonance data for *N*-ethylhexylone (**2**).

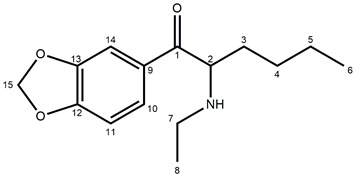		
Atom Position	Carbon Chemical Shifts (ppm)	Proton Chemical Shifts (ppm)
1	194.60	–
2	60.62	5.20 (t, *J* = 5.5 Hz, 1H)
3	30.22	1.89 (m, 2H)
4	26.11	1,19 (m, 2H)
5	22.34	1.04 (m, 2H)
6	11.68	0.76 (t, *J* = 7.2 Hz, 3H)
7	41.65	2.88 (m, 1H); 2.99 (m, 1H)
8	13.99	1.24 (t, *J* = 7.3 Hz, 3H)
9	128.91	–
10	126.41	7.76 (dd, *J* = 8.2; 1.8 Hz, 1H)
11	109.03	7.13 (d, *J* = 8.2 Hz, 1H)
12	153.40	–
13	148.73	–
14	108.26	7.57 (d, *J* = 1.7 Hz, 1H)
15	103.02	6.20 (s, 2H)
N-H	–	8.96 (bs, 1H); 9.28 (bs, 1H)

## Data Availability

The data presented in this study are available upon request from the corresponding authors.

## Supplementary Materials

The following supporting information can be downloaded at https://www.mdpi.com/article/10.3390/molecules30102182/s1. Figure S1. ESI-MS/MS spectrum of *N*-butyl-norbutylone (**1**); Figure S2. ESI-MS/MS spectrum of *N*-ethylhexylone (**2**); Figure S3. ^1^H-^1^H NMR spectrum of *N*-butyl-norbutylone (**1**); Figure S4. ^1^H-^1^H NMR spectrum of *N*-ethylhexylone (**2**).
