# Supplementary material for: Spectroscopic and Chromatographic Characterization of Two Isomeric Cathinone Derivatives: N-Butyl-Norbutylone and N-Ethylhexylone
Source: Molecules. 2025 May 16;30(10):2182. doi: 10.3390/molecules30102182 (PMC12114368; doi:10.3390/molecules30102182)
Supplement: Supplementary file 1 [file molecules-30-02182-s001.zip › molecules-3637844-supplementary.pdf]

Supplementary Materials

Spectroscopic and Chromatographic Characterization of Two Isomeric Cathinone Derivatives: *N*-butyl-norbutylone and *N*-ethylhexylone

Marcin Rojkiewicz [1,*], Piotr Kuś [1], Josef Jampilek [1,2], Andrzej Bąk [1] and Violetta Kozik [1,*]

1 Institute of Chemistry, University of Silesia, 9 Szkolna Street, 40-006 Katowice, Poland; pkus@ich.us.edu.pl (P.K.); josef.jampilek@gmail.com (J.J.); andrzej.bak@us.edu.pl (A.B.)

2 Department of Analytical Chemistry, Faculty of Natural Sciences, Comenius University, Ilkovicova 6,
842 15 Bratislava, Slovakia

* Correspondence: marcin.rojkiewicz@us.edu.pl (M.R.); violetta.kozik@us.edu.pl (V.K.)

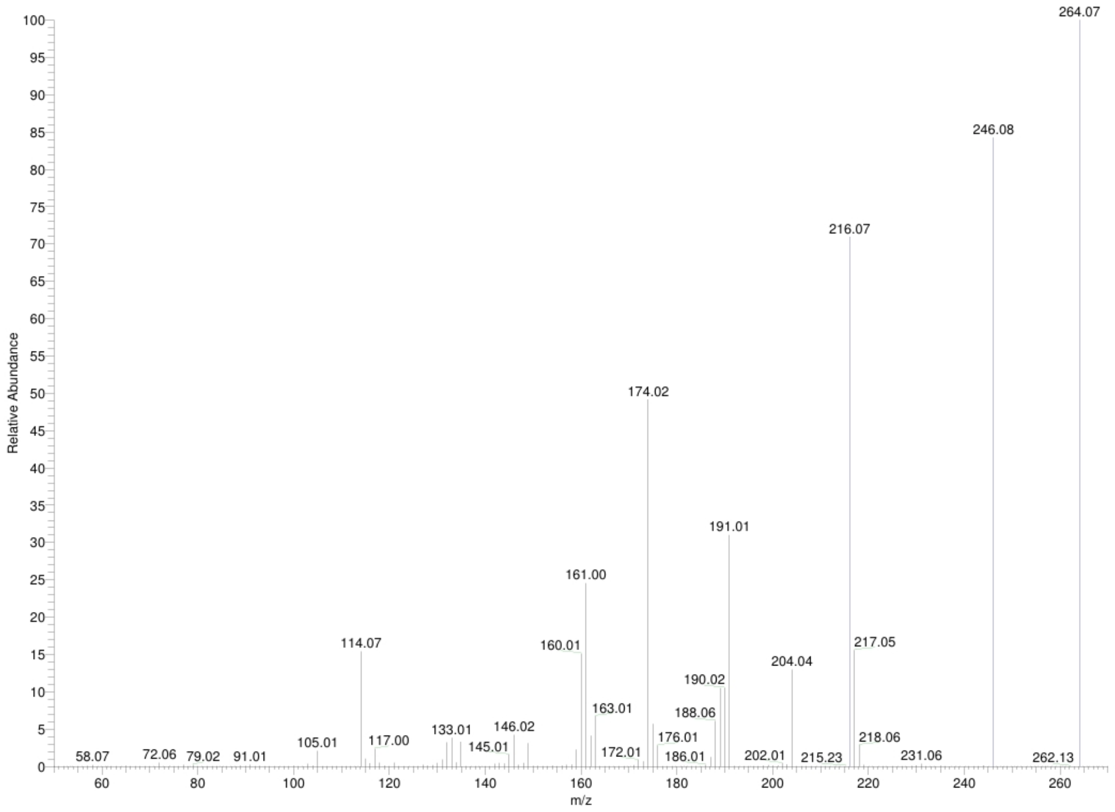

Figure S1. ESI-MS/MS spectrum of *N*-butyl-norbutylone (**1**).

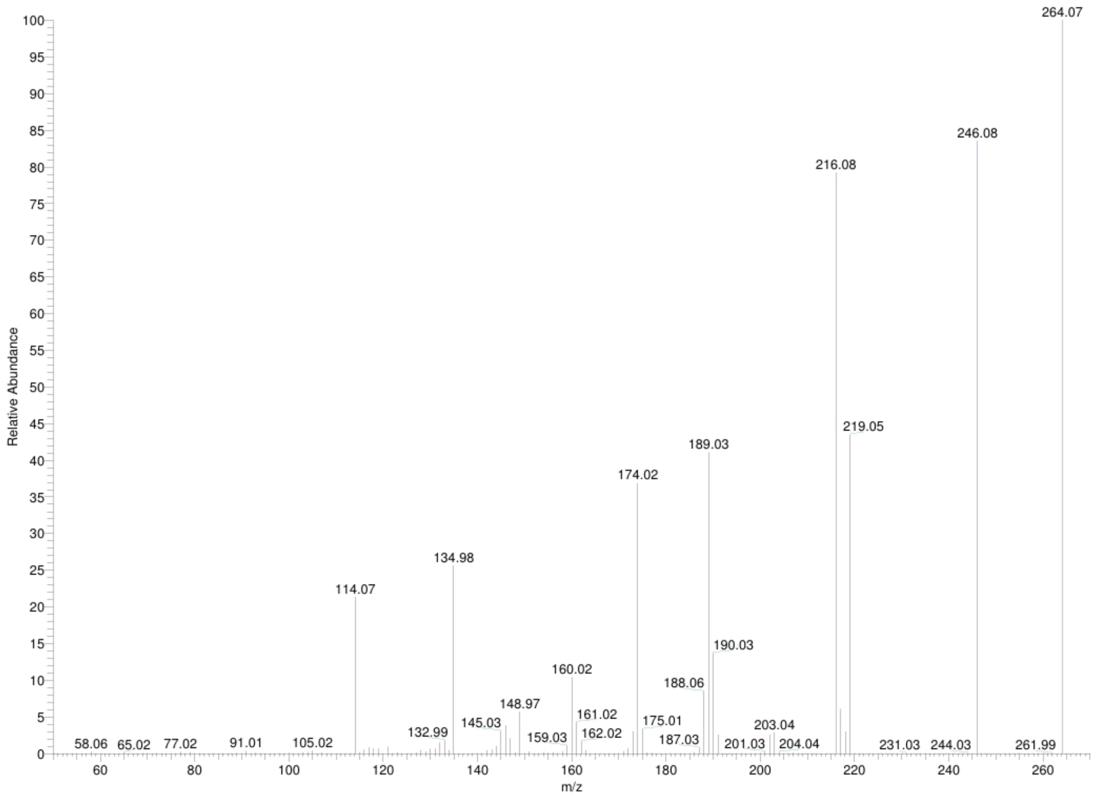

Figure S2. ESI-MS/MS spectrum of *N*-ethylhexylone (**2**).

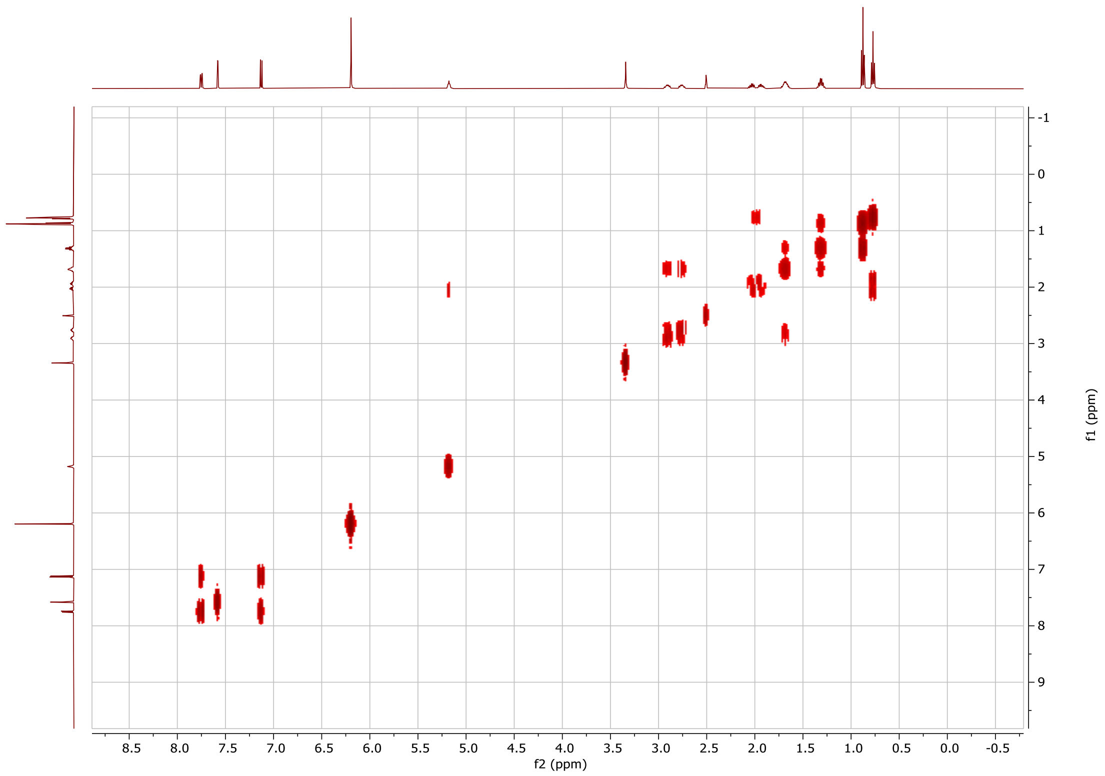

Figure S3. ^{1}H-^{1}H NMR spectrum of *N*-butyl-norbutylone (**1**).

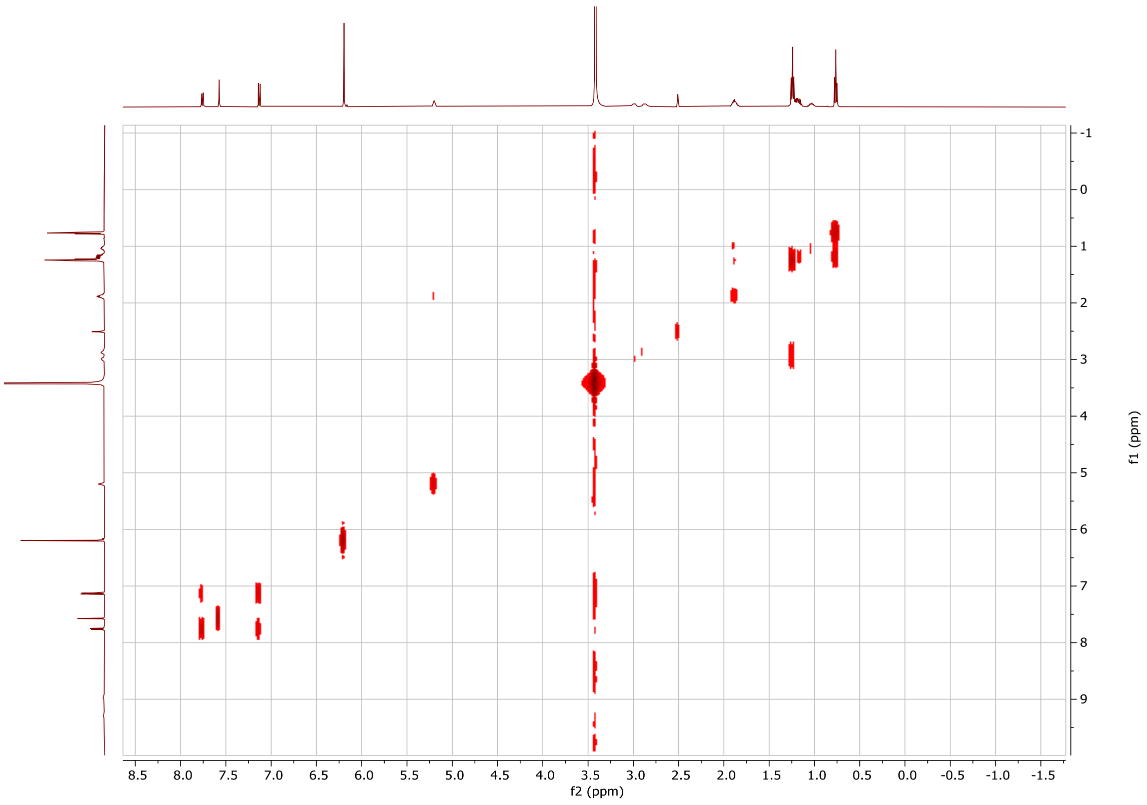

Figure S4. ^{1}H-^{1}H NMR spectrum of *N*-ethylhexylone (**2**).
